# Understanding the adoption and use of point-of-care tests in Dutch general practices using multi-criteria decision analysis

**DOI:** 10.1186/s12875-018-0893-4

**Published:** 2019-01-10

**Authors:** Michelle M. A. Kip, J. Marjan Hummel, Elra B. Eppink, Hendrik Koffijberg, Rogier M. Hopstaken, Maarten J. IJzerman, Ron Kusters

**Affiliations:** 1 Department of Health Technology and Services Research, Faculty of Behavioural, Management and Social Sciences, Technical Medical Centre, University of Twente, P.O. Box 217, 7500 AE Enschede, The Netherlands; 2 Star-SHL diagnostic center, Etten-Leur, The Netherlands; 3 Laboratory for Clinical Chemistry and Haematology, Jeroen Bosch Ziekenhuis, Den Bosch, The Netherlands

**Keywords:** Analytic hierarchy process, Health technology assessment, Adoption, Multi-criteria decision analysis, Point-of-care tests, Preference elicitation, Primary care

## Abstract

**Background:**

The increasing number of available point-of-care (POC) tests challenges clinicians regarding decisions on which tests to use, how to efficiently use them, and how to interpret the results. Although POC tests may offer benefits in terms of low turn-around-time, improved patient’s satisfaction, and health outcomes, only few are actually used in clinical practice. Therefore, this study aims to identify which criteria are, in general, important in the decision to implement a POC test, and to determine their weight. Two POC tests available for use in Dutch general practices (i.e. the C-reactive protein (CRP) test and the glycated haemoglobin (HbA_1c_) test) serve as case studies. The information obtained from this study can be used to guide POC test development and their introduction in clinical practice.

**Methods:**

Relevant criteria were identified based on a literature review and semi-structured interviews with twelve experts in the field. Subsequently, the criteria were clustered in four groups (i.e. user, organization, clinical value, and socio-political context) and the relative importance of each criterion was determined by calculating geometric means as implemented in the Analytic Hierarchy Process. Of these twelve experts, ten participated in a facilitated group session, in which their priorities regarding both POC tests (compared to central laboratory testing) were elicited.

**Results:**

Of 20 criteria in four clusters, the test’s clinical utility, its technical performance, and risks (associated with the treatment decision based on the test result) were considered most important for using a POC test, with relative weights of 22.2, 12.6 and 8.5%, respectively. Overall, the experts preferred the POC CRP test over its laboratory equivalent, whereas they did not prefer the POC HbA_1c_ test. This difference was mainly explained by their strong preference for the POC CRP test with regard to the subcriterion ‘clinical utility’.

**Conclusions:**

The list of identified criteria, and the insights in their relative impact on successful implementation of POC tests, may facilitate implementation and use of existing POC tests in clinical practice. In addition, having experts score new POC tests on these criteria, provides developers with specific recommendations on how to increase the probability of successful implementation and use.

**Electronic supplementary material:**

The online version of this article (10.1186/s12875-018-0893-4) contains supplementary material, which is available to authorized users.

## Background

In the last decades, the use of diagnostic tests has increased rapidly. This number is expected to rise even further given the increasing number of people with multiple (chronic) conditions, and the availability of a wide range of biomarkers for monitoring disease and treatment response [1, 2]. Rapid, accurate diagnostic tests have the potential to improve targeted treatments or referrals and thereby improve the overall quality and efficiency of care delivery. However, the availability of new diagnostic tests also challenges physicians to effectively use and interpret the (combination of) test results [3]. This particularly applies to general practitioners (GPs), as they encounter a wide variety of medical conditions among patients presenting in their general practice, and have to decide on whether or not (and which) diagnostic test(s), if any, to perform in those patients. Although most of the laboratory tests requested by GPs are still performed in central laboratories, advances in technology increasingly allow to perform some of these tests directly during consultation, i.e. at the point-of-care [4], offering large benefits in terms of timely and targeted treatment [5].

More specifically, POC tests have been shown to improve patient’s satisfaction, treatment adherence, and health outcomes, while also saving time and costs [4, 6–10]. In addition, previous research indicated that GPs express the desire to have POC tests available to help them diagnose a range of acute and chronic conditions [11]. Despite these clear advantages, only a limited number of POC tests are actually used in clinical practice [11, 12]. Reasons for non-use include concerns about test accuracy, over-reliance on test results, undermining clinical judgment, limited added value on top of clinical judgement, as well as higher average costs of POC testing compared to central laboratory testing, and test reimbursement [12, 13].

The decision to actually implement a POC test instead of continuing to request central laboratory tests is a tradeoff between multiple, often conflicting, objectives [14–16]. The use of structured, explicit approaches to decision-making involving multiple criteria can improve the quality of decisions and identify factors for improvement [16]. A set of techniques known as multiple criteria decision analysis (MCDA) can be used for this purpose [16]. In health technology assessment, MCDA can be used to obtain clarity on the relevance of decision criteria, and on the performance of alternative technologies (e.g. diagnostic tests) on these criteria, thereby increasing the consistency, transparency, and legitimacy of ensuing implementation decisions [16]. One of the most frequently applied techniques for MCDA, which is often applied in group decision making, is the analytic hierarchy process (AHP) [17, 18]. In AHP, complex decision problems are reduced to multiple pairwise comparisons, and captures both subjective and objective criteria in the decision-making process [17]. Besides its application in healthcare, AHP has been applied to a large variety of other decision problems, ranging from decision problems in industry and politics to the composition of sports teams [19].

The current study uses AHP to estimate the relative preference for a POC test as compared to the same test when performed in a central laboratory. This will give insights in the criteria which are considered most important in the decision on whether or not to implement and use a POC test. We selected two cases that impact care delivery yet face implementation difficulty, i.e. the POC C-reactive protein (CRP) test, and the POC glycosylated haemoglobin (HbA_1c_) test. CRP is an acute-phase protein measured in a patient’s blood enabling the physician to differentiate between patients with bronchitis from those with community-acquired pneumonia. Use of the POC tests allows GPs to immediately decide whether or not antibiotic treatment is required [10, 20–24], and was used by 80% of Dutch GPs in 2015 [25].

The HbA_1c_ test is used to regularly monitor diabetes patients. Those patients typically have to visit a nurse or phlebotomist for a venipuncture, 1–2 weeks prior to their appointment [26]. A POC HbA_1c_ blood test offers immediate test results, thereby allowing immediate therapeutic decision making, and consequently, reducing patient visits for phlebotomies [26, 27]. In 2015, the POC HbA_1c_ test was used by 19% of Dutch GPs [25].

Although the abovementioned examples indicate the potential added value of both POC tests, they differ strongly in their degree of implementation. Therefore, these tests were selected as case studies to get insight into the relative preference of experts regarding the use of POC tests in primary care. This preference is estimated using AHP, by identifying and weighting the criteria that are relevant to the decision to use the POC test. Thereby, this study aims to determine which criteria are, in general, important in the decision to implement and use a POC test in clinical practice. This information is highly relevant to all stakeholders involved in the process of developing, evaluating and implementing POC tests in clinical practice.

## Methods

### Multi-criteria decision analysis with the analytic hierarchy process

**Fig. 1:**
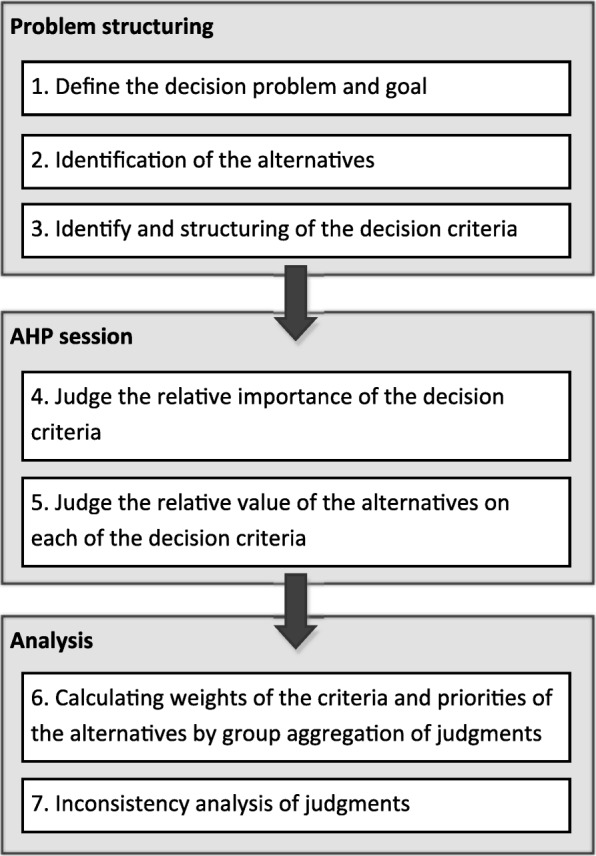
Steps in the AHP. This figure shows an overview of the different steps that are performed in the analytical hierarchy process (AHP)

The implementation of an MCDA study follows several separate steps [18], and guidance is provided by the taskforce of the International Society For Pharmacoeconomics and Outcomes Research [28]. Firstly, the framework for the analysis is determined by setting the goal of the analysis, identifying the alternatives that are compared (i.e. POC CRP and POC HbA_1c_ as alternatives to their equivalent central laboratory tests), and defining the relevant criteria. Secondly, importance weights are obtained for each of the criteria. Thirdly, the performance of both POC tests on each decision criterion was valued relatively to the performance of their laboratory equivalents. The final step is the aggregation of the scores and weights to determine the relative value for the POC CRP and the POC HbA_1c_ test, as alternative to their equivalent central laboratory test. An overview of these steps, which will be referred to in the remainder of the methods section, is provided in Fig. 1. We assume that the technique with the highest value is the preferred one, and is therefore most likely to be implemented (and used) in clinical practice.

### Problem structuring

#### Defining the decision problem and goal & identification of the alternatives (steps 1 & 2)

For this AHP, it is assumed that the use of a POC CRP test or a POC HbA_1c_ test will replace the use of the equivalent test performed in a central laboratory, instead of being used as an additional test in the diagnostic track. This means that the criteria regarding the (potential) use of a POC test within the GP’s office will be compared to the criteria of the equivalent test when performed in a central laboratory (step 1) based on two case studies (i.e. CRP and HbA_1c_) (step 2).

Besides the difference in the degree of diffusion of the POC CRP and the POC HbA_1c_ test (as mentioned in the introduction), both cases have very different areas of application, thereby increasing the probability that the results obtained from this AHP are also relevant to the wide range of POC tests that can be used in Dutch general practices.

#### Identification and structuring of the decision criteria (step 3)

Relevant criteria for the AHP analysis were identified from a literature search, of which the details are described in Additional file 1. The literature search resulted in the inclusion of 7 journal articles, 1 guideline, and 3 reports [9, 11, 12, 29–36]. Each paper was independently assessed by two reviewers (EE and MK) to identify all relevant criteria. The criteria obtained were compared to a previously published study in implementation science [37]. As this previous study specifically focused on guidelines and intervention programs in healthcare [37], the criteria identified from the current literature search differed from those reported in this previous study. However, this previous study categorized all criteria into four groups of main criteria, which are: 1) the user, 2) the organization, 3) the clinical value, and 4) the socio-political context [37]. As these generic groups are considered applicable to all kind of health-related interventions, this categorization of criteria was used for the purpose of the current study. Both the identification of the criteria, as well as categorizing those criteria into the four main groups of criteria were performed by the same two reviewers (EE and MK). Differences were discussed until consensus was reached.

##### Expert panel

In order to judge both the validity and completeness of this list of criteria, the (potential) presence of redundant or overlapping criteria, as well as the preferential independence of the criteria [28], the initial list and suggested structure were validated by means of individual interviews with 12 experts. In addition, these experts also judged the clarity, completeness, and unambiguity of the *definitions* accompanying each of the criteria. The panel of experts was selected in such a way to represent all stakeholders who are either involved in the process of POC test development, or its implementation and use in clinical practice. A full overview of the participating experts and their professional backgrounds is presented in Table 1. All participating experts were informed on the goal of the study as well as on the duration of the interview and the AHP session. As the current study focused on the Dutch setting, all selected participants were living and employed in the Netherlands. The participants were initially approached via email. Participation was voluntary and the experts had the opportunity to quit participation at any time. This study was approved by the Ethics Committee of the faculty Behavioural, Management and Social Sciences of the University of Twente, Enschede, the Netherlands.

**Table 1:** Composition of expert team

No.	Profession	Core relation to POC testing in general practices
1	GP	User of CRP and HbA_1c_ test
2	GP	User of CRP test, non-user HbA_1c_ test
3	GP	User of CRP test, non-user HbA_1c_ test
4	GP’s assistant	User of CRP test, non-user HbA_1c_ test, former nurse
5	Diabetic patient	User of HbA_1c_ test (as a patient), biology teacher (familiar with CRP)
6	Clinical chemist	Laboratory professional, specialized in POC tests in primary care
7^b^	Clinical chemist	Laboratory professional, specialized in POC tests in primary care
8	Technology developer	Director lab-on-a-chip company
9	Policy maker	Concerned with the quality of care provided in primary care
10^a^	POC specialist	Expert in POC testing, GP
11^a^	Payer	Insurer in primary health care, former GP
12^b^	Payer	Former director of health insurance company, and professor in healthcare

^a^*did not participate in the group AHP session but completed the AHP session afterwards.* ^b^*did not participate in the AHP session. CRP = C-reactive protein, GP = general practitioner, HbA*_*1c*_ *=* glycated haemoglobin, *POC = point-of-care*

**Fig. 2:**
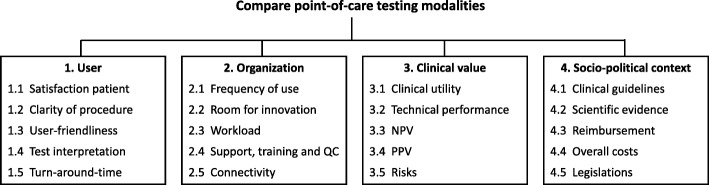
Hierarchical structure of the AHP. This figure shows an overview of the hierarchical structured used for the analytical hierarchy process (AHP). NPV = negative predictive value, POC = point-of-care, PPV = positive predictive value, QC = quality control

Validation of the list of criteria obtained from literature was performed by means of individual, semi-structured interviews with each of the 12 experts (by EE), either via phone or at the expert’s workplace. After each session, the suggested adaptations to the identification and structuring of the criteria, were discussed by MK and EE until consensus was reached. The updated list was then used as input for the next semi-structured interview. This repetitive process resulted in a final list of 20 criteria (categorized in four groups of five subcriteria), which were expected to affect the implementation and use of POC tests in general practices. These criteria were used to compose the hierarchical evaluation structure (Fig. 2). A full description of the 20 subcriteria, and the accompanying range applied in the AHP session, is included in Additional file 2 (step 3).

All 12 experts who participated in the initial interviews (Table 1), were also invited to participate in the AHP session (at the University of Twente). A group setting was chosen for this AHP because it allows panel members to share information about their attitudes, beliefs, as well as knowledge, which underlie the priorities they assign to the outcome measures [38]. One clinical chemist (expert no. 7) and the former director of a health insurance company (expert no. 12) were however unable to participate in the AHP session. All remaining ten experts agreed to participate in the AHP session, although two of those experts (expert no. 10 and expert no. 11) were unable to attend the actual group session, and therefore completed the AHP session individually afterwards.

### AHP session (steps 4 & 5)

The AHP session was performed in accordance with previously published literature and/or guidance documents [18, 39]. A three-hour AHP session was organized (by EE, and with attendance of JH and MK) during which the expert team discussed the relative importance of the four main criteria as well as each of the five (sub) criteria within each main criterion. This session was performed using Team Expert Choice software (Expert Choice, Arlington, VA), a group decision support system which incorporates the mathematical procedures of AHP, and all conversations during this AHP session were audio recorded.

During this session, all members of the expert team were asked to provide their judgments on pairwise comparisons of the importance of the four main criteria, as well as the four groups of five subcriteria (step 4). In this step, the relative importance of the main criteria are judged using a nine-point rating scale. Criteria that are judged as equally important will receive a score of ‘1’. If one of the criteria is judged more important than the other, the more important criterion will receive a score between 2 (equally to moderately more important), and 9 (extremely more important) [18]. Subsequently, the importance of each pair of subcriteria (stemming from the main criterion) is compared. Likewise, the preferences for the selected alternatives (i.e. for POC CRP vs. central laboratory CRP, and for POC HbA_1c_ vs. central laboratory HbA_1c_) with regard to the 20 criteria, is determined on similar nine-point scales (step 5). The performance of both POC tests and the central laboratory tests on the selected criteria was left to the experts’ judgement. Responses were collected individually using hand-held remote controlled keypads. All individual judgments were projected on a screen, allowing the expert team members to provide their motivation for their individual scores, and to share their expertise on this topic. To allow the other team members to incorporate the expertise and motivations that were shared, they had the opportunity to alter their judgements during those dialogs. A detailed overview of the AHP session, and the pairwise-comparisons made, is provided in Additional file 2.

### Analysis (steps 6 & 7)

For each pairwise comparison, the final individual judgments were aggregated, based on the geometric mean. This resulted in group weighing factors which represented the relative importance of each of the criteria, and these weighing factors were used to calculate the relative preference for the alternatives (i.e., the POC test or the central laboratory test) (step 6). In addition, the inconsistency of the expert’s judgments was calculated after the pair-wise comparisons of each of the main criteria, and after each of the 20 subcriteria. In accordance with literature, an inconsistency below 0.1 was considered acceptable [17]. In case of a too high inconsistency, the experts were asked to reconsider the pair-wise comparison which caused this inconsistency (to make sure that the criteria compared were well-understood), or they were asked to fill in an additional comparison (step 7).

## Results

The results of the pairwise comparisons of the four main criteria and the 20 subcriteria, as well as the preferences regarding the POC CRP and POC HbA_1c_ test (as compared to their laboratory equivalents) are shown in Table 2.

**Table 2:** Result of pairwise comparisons

Determinant		Weights*		Performance scores			
		Weight of criterion	Overall weight	POC CRP	CRP central lab	POC HbA_1c_	HbA_1c_ central lab
Determinants in relation to the user		13.6%	NA	81.9%	18.1%	68.2%	31.8%
1	Satisfaction patient	25.4%	3.5%	89.9%	10.1%	85.3%	14.7%
2	Clarity of procedure	24.8%	3.4%	71.3%	28.7%	56.1%	43.9%
3	User-friendliness	21.0%	2.9%	84.2%	15.8%	78.1%	21.9%
4	Test interpretation	6.4%	0.9%	69.0%	31.0%	50.0%	50.0%
5	Turn-around-time	22.3%	3.0%	90.0%	10.0%	89.8%	10.2%
Determinants in relation to the organisation		20.9%	NA	65.4%	34.6%	48.8%	51.2%
6	Frequency of use	14.6%	3.1%	88.6%	11.4%	49.5%	50.5%
7	Room for innovation	33.5%	7.0%	89.7%	10.3%	85.0%	15.0%
8	Workload	22.1%	4.6%	77.1%	22.9%	29.6%	70.4%
9	Support, training and quality control	25.4%	5.3%	21.3%	78.7%	24.4%	75.6%
10	Connectivity	4.4%	0.9%	35.7%	64.3%	46.5%	53.5%
Determinants in relation to the clinical value		51.8%	NA	55.1%	44.9%	45.8%	54.2%
11	Clinical utility	42.8%	22.2%	89.4%	10.6%	53.5%	46.5%
12	Technical performance	24.4%	12.6%	28.1%	71.9%	33.5%	66.5%
13	Negative Predictive Value	13.7%	7.1%	48.3%	51.7%	50.0%	50.0%
14	Positive Predictive Value	2.8%	1.5%	48.3%	51.7%	50.0%	50.0%
15	Risks	16.4%	8.5%	34.4%	65.6%	33.5%	66.5%
Determinants in relation to the socio-political context		13.8%	NA	72.9%	27.1%	49.1%	50.9%
16	Clinical guidelines	34.1%	4.7%	82.1%	17.9%	47.3%	52.7%
17	Scientific evidence	23.7%	3.3%	85.0%	15.0%	60.5%	39.5%
18	Reimbursement	28.5%	3.9%	60.7%	39.3%	42.2%	57.8%
19	Overall costs	8.3%	1.1%	88.8%	11.2%	55.5%	44.5%
20	Legislations	5.5%	0.8%	42.3%	57.7%	40.5%	59.5%
Overall preference for POC or central laboratory test				62.9%	37.1%	49.4%	50.6%

This table shows the results of the pairwise comparisons of the four main criteria and the 20 subcriteria, as well as the preferences regarding the POC CRP and POC HbA_1c_ test (as compared to their central laboratory equivalents). The overall weight is obtained by multiplying the weight of the main criterion which each of the subcriteria. The definition of each of the criteria is provided in Additional file 3. *NA* not applicable, *POC* point-of-care. * The sum of columns may not add up to 100.0% due to rounding

Table 2 indicates that a change in the main criterion ‘clinical value’, as caused by the use of a POC test, is estimated to be most important for comparing preferences for a POC test with its central laboratory test. The other main criteria in order of decreasing importance are ‘organisation’, ‘socio-political context’ and ‘user’. An overall group inconsistency within those four main criteria of 0.05 was found, which is acceptable considering the 0.1 threshold (Additional file 3).

**Fig. 3:**
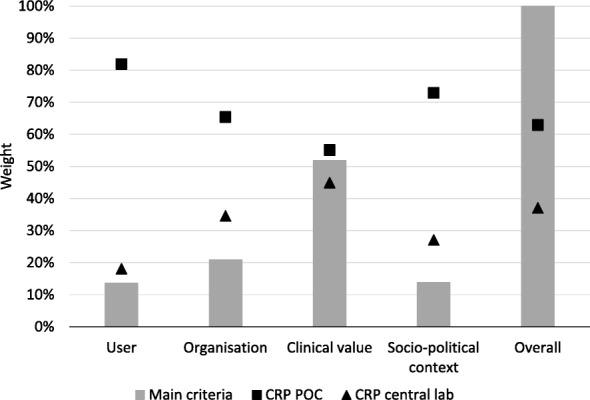
Result of AHP analysis on the POC CRP test as compared to central laboratory testing. This figure shows the result of the analytical hierarchy process (AHP) analysis on the POC CRP test as compared to using the central laboratory test, on the four main criteria and the overall result, as well as the performance of the two tests on each criterion. The grey bars represent the relative weights of the four main criteria. The figure represents the performance of the POC test (square) and lab-test (triangle) on each criterion. CRP = C-reactive protein, POC = point-of-care

**Fig. 4:**
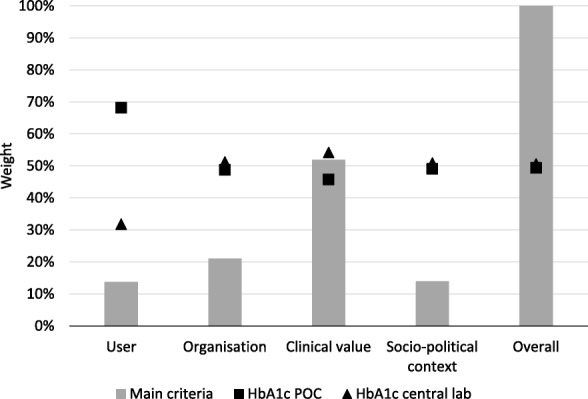
Result of AHP analysis on the POC HbA_1c_ test as compared to central laboratory testing. This figure shows the result of the analytical hierarchy process (AHP) analysis on the POC HbA_1c_ test as compared to using the central laboratory test, the four main criteria and the overall result, as well as the performance of the two tests on each criterion. The grey bars represent the relative weights of the four main criteria. The figure represents the performance of the POC test (square) and lab-test (triangle) on each criterion. HbA_1c_ = glycated hemoglobin, POC = point-of-care

Among the 20 subcriteria, the results indicate that the subcriterion ‘clinical utility’ is expected to have the highest impact on the relative preference for the POC test as compared to the central laboratory test (relative impact 22.2%), followed by ‘technical performance’ (12.6%), and ‘risks’ (8.5%). The high weights of those three subcriteria can also be explained by the high weight of the main criterion ‘clinical value’ (51.8%), to which those three subcriteria belong. The subcriteria ‘legislations’, ‘connectivity’, and ‘test interpretation’ were however found to have very little impact. The results of the comparison of the preferences of using the POC CRP test and the POC HbA_1c_ test as compared to using the equivalent central laboratory tests, are shown in Figs. 3 and 4, respectively.

Results indicate that the experts strongly prefer the POC CRP test as compared to its central laboratory test over all four main criteria, resulting in an overall preference of 62.9% vs. 37.1%. The top four subcriteria that determined this preference involved the expected shorter ‘turn-around time’, an expected increase in ‘patient satisfaction’, the ‘room for innovation’ that is experienced, and its expected improvement in ‘clinical utility’ (Table 2 and Additional file 3).

However, the overall preference for the POC CRP test (over its central laboratory test) is dependent on the weights assigned to each of the subcriteria. Results indicate that the subcriteria ‘clinical utility’, and ‘room for innovation’ are most influential in favoring the POC test, whereas the subcriterion ‘technical performance’ was most influential in favoring the laboratory test.

In contrast, for the HbA_1c_ test, the experts displayed (almost) equal preference for the POC and the central laboratory test (i.e. 49.4% vs. 50.6%). More specifically, with regard to the groups of main criteria, the POC test is only preferred over its central laboratory test for the main criterion ‘user’, caused by the preference for the POC HbA_1c_ test with regard to the subcriteria ‘satisfaction patient’, ‘user-friendliness’, and ‘turn-around-time’ (Table 2 and Additional file 3). When taking into account the weight of the subcriteria, the subcriterion ‘room for innovation’ was found to be most influential in favor of POC HbA_1c_. However, in terms of ‘clinical value’, which is the main criterion with the highest relative weight, the central laboratory test is slightly preferred over the POC test. This is mainly caused by the expected decrease in ‘technical performance’ and expected increase in ‘risks’ related to making management decision based on the POC test result (Table 2 and Additional file 3). These lower scores could not be offset by a slightly higher score on the subcriterion ‘clinical utility’ (i.e. 53.5% vs. 46.5%), even though this is the subcriterion with the highest relative weight.

When considering the differences in preferences assigned to the 20 subcriteria for the POC and the central laboratory test (for both CRP and HbA_1c_), the main differences were found in the subcriteria ‘clinical utility’, the user’s ‘workload’, its expected ‘frequency of use’, the extent to which the test is incorporated in current ‘clinical guidelines’, as well as its impact on ‘overall costs’.

## Discussion

The results indicate that the expert panel considered the main criterion ‘clinical value’ most important for comparing preferences for a POC test to its central laboratory equivalent (i.e. a relative weight of 51.8%). In addition, its subcriterion ‘clinical utility’ was assigned the highest relative weight of all 20 subcriteria (i.e. 22.2%).

When considering the overall outcome of the two case studies, POC CRP was preferred over its laboratory equivalent, whereas POC HbA_1c_ was not. Specifically, the POC CRP test was strongly preferred with regard to the subcriterion ‘clinical utility’, as opposed to HbA_1c_. This is in line with previous research, concluding that GPs prefer to have POC tests for rapidly diagnosing (or excluding) acute and/or serious conditions [11, 32]. In addition, the POC CRP test may be used for multiple clinical indications [40], in contrast to POC HbA_1c_ [41, 42]. Also, the overall preference for POC CRP testing is in line with its current use (i.e. 80% of Dutch GPs in 2015) [25]. In 2013, this percentage was however only 48% [11]. As the expert panel assigned a high preference for POC CRP with regard to the subcriterion ‘clinical guidelines’, the increased use of (POC) CRP testing is likely explained by its uptake in Dutch guidelines [43].

Furthermore, with regard to the subcriteria ‘turn-around-time’ and ‘patient satisfaction’, results indicate a strong preference for *both* POC CRP and POC HbA_1c_. Similar findings have been reported in literature [4, 6]. For POC HbA_1c_, these preferences are most likely attributable to the fact that this tests prevents an additional patient visit for a phlebotomy [26]. However, as the POC HbA_1c_ test is intended to be performed during the actual GP consultation, the experts assume that it will negatively affect the GP’s ‘workload’. In addition, previous research indicates that not all currently available devices meet the minimum performance requirements [44, 45], and cost-effectiveness analyses have been inconclusive [27, 46], which explains why this test is not considered of much added value in terms of ‘technical performance’ and the impact on ‘overall costs’. This is in line with previous studies on POC testing (in general) [12, 13].

Interestingly, results indicate that the expert panel considered the test’s NPV to be far more important than its PPV (i.e. overall weight 7.1% vs. 1.5%), independent of the type of test that was evaluated. This difference is most likely attributable to the strong gatekeeping function of GPs in the Netherlands. Consequently, diagnostic tests for use in primary care are primarily aimed at having a high NPV (to rule out a (serious) condition), instead of a high PPV (as is required for ruling in) [36].

### Strengths and limitations

The main strength of the current study is that the set of criteria selected from literature were validated in interviews with a multidisciplinary team of 12 experts. This most likely contributed to preventing the AHP structure from becoming too complex by categorizing all aspects into a list of 20 subcriteria, while simultaneously ensuring that all relevant aspects remain incorporated.

As the GP is a key stakeholder in the decision to implement and use a POC test, a strength of this study is that three GPs participated both in the expert panel and the AHP session. As these GPs differed in the number of years working experience and/or had a different viewpoint on using POC tests, this likely resulted in a representative weighting of the criteria. However, this overrepresentation of GPs may have led to confirmation bias [47], as their current POC test use (i.e. one GP used POC HbA_1c_, whereas all three GPs used POC CRP) may have strengthened their preference towards POC CRP. When performing a separate analysis in which only the GPs’ opinions were taken into account, it was indeed observed that the overall preference for a POC test increased from 62.9 to 69.9% for CRP, and from 49.4 to 51.2% for HbA_1c_ (Additional file 3: Table S3a). However, the overall conclusion was unaffected.

As the health insurer and the POC expert were not able to attend the group AHP session, they completed it individually afterwards. Both experts were provided with the scores (and explanations) given during the group session. Consequently, it cannot be ruled out that the scores of the other experts would have been affected if those two experts would have attended the group session. However, subgroup analyses revealed that the overall preference for a POC test was unaffected when the scores of these two experts were excluded from the analyses (i.e. 61.6% vs. 62.9% for POC CRP; 48.4% vs. 49.4% for POC HbA_1C_, as shown in Additional file 3: Table S3b).

In the current study, preference weights have been assigned to the four main criteria, as well as to the five subcriteria belonging to each of these four groups. The risk of such a clustered evaluation is that the weight of a subcriterion depends on the weight assigned to its main criterion. To illustrate this, the subcriterion ‘clinical guidelines’ was assigned a weight of 34.1%, but as the main criterion it belongs to (i.e. ‘socio-political context’) was only assigned a weight of 13.8%, the overall weight of ‘clinical guidelines’ is only 4.7%. This may result in (slightly) underestimating the overall weight of some important criteria. The main benefit of this clustered evaluation, however, is that bias of overweighting main criteria is avoided, which would occur when weights of partially overlapping subcriteria were summed to calculate the weights of the main criteria.

### Recommendations

Although the strong differences in both the degree of implementation as well as in the application of the CRP and the HbA_1c_ test likely enhance the generalizability of the study’s results, future AHP sessions are recommended to find out whether this single set of criterion weights can reliably be applied to a wide variety of tests and settings.

Although the results of the current study indicate a preference for CRP testing on a POC analyzer, this cannot guarantee its (further) implementation and use in clinical practice. To make such a decision, additional (temporal) costs (and efforts) related to switching to a POC test should be considered. This for example includes costs related to the purchase and maintenance of the POC testing device, to obtaining the (blood) sample, as well as to educating the test’s users. In addition, the results indicate that, besides costs, many other factors play a role in decisions regarding test implementation and use. As many of these factors cannot be captured in conventional cost-effectiveness analyses, it is important to obtain insight in stakeholders’ experiences and preferences early on during the process of (POC) test development and its (eventual) implementation in clinical practice.

## Conclusion

In conclusion, the list of criteria identified in the current study may facilitate efficient implementation and use of existing POC tests. In addition, it is likely valuable to predict the likelihood of implementation and use of POC tests in early stages of development. The insights obtained in the barriers and facilitating factors of POC tests can be used to either predict the likelihood of implementation and use of POC tests in early stages of development, or to increase this probability, by allowing test developers to focus on the criteria that were considered most important.

## Additional files

Additional file 1: Literature search. This file contains the extensive description of the literature search, which was used to identify criteria relevant for the analytical hierarchy process. (PDF 103 kb)

Additional file 2: Questions used for semi-structured interviews, and design of analytical hierarchy process session. This file contains an overview of the interview questions used for the semi-structured interviews, as well as an overview of the design of the analytical hierarchy process session. (PDF 291 kb)

Additional file 3: Results of interviews and analytical hierarchy process. This supplementary file contains an overview of the detailed results of the analysis of the analytical hierarchy process (AHP), including the weighting of the subcriteria, the group inconsistency and missing judgements during the analytical hierarchy process session, as well as the preferences regarding the subcriteria for the two alternatives (i.e. point-of-care (POC) C-reactive protein (CRP) vs. CRP in a central laboratory, as well as POC glycated haemoglobin (HbA1c) vs. HbA1c in a central laboratory). (DOCX 73 kb)

## Abbreviations

AHP: Analytic hierarchy process
CRP: C-reactive protein
GP: General practitioner
HbA_1c_: Glycated haemoglobin
MCDA: Multi-criteria decision analysis
POC: Point-of-care
