# Supplementary material for: Understanding the adoption and use of point-of-care tests in Dutch general practices using multi-criteria decision analysis
Source: BMC Fam Pract. 2019 Jan 10;20:8. doi: 10.1186/s12875-018-0893-4 (PMC6327588; doi:10.1186/s12875-018-0893-4)
Supplement: Supplementary file 1 — Literature search. This file contains the extensive description of the literature search, which was used to identify criteria relevant for the analytical hierarchy process. (PDF 103 kb) [file 12875_2018_893_MOESM1_ESM.pdf]

Additional file 1: literature search

A literature search was performed in Scopus and PubMed, using combinations of the following search terms: *point-of-care testing* OR *point of care testing* OR *point-of-care test* OR *point of care test* OR *POC* OR *POC test* OR *POCT*, *general practitioner* OR *GP* OR *general practice, innovation, healthcare* OR *health care*, *implementation*, *measurement instrument* OR *factors* OR *determinants*, and *innovation*. Potentially relevant articles were selected based on title and abstract, and the full text of the remaining articles was assessed to identify relevant articles. Only articles published in English or Dutch, and available from the university library or open access databases, were included. In addition, a search on the internet, using similar search terms, was conducted to obtain general information (like guidelines and reports), which could not be obtained via PubMed or Scopus. The literature search resulted in the inclusion of 7 journal articles, 1 guideline, and 3 reports (1-11). The literature search was performed in September 2015.

References:

1. Kleinveld HA, Raijmakers MTM, Vermeer HJ, Oosterhuis WP. Visiedocument nvkc point-of-care testen in de eerste lijn. Vol. Heerlen, 2012:8.
2. Briggs C, Kimber S, Green L. Where are we at with point-of-care testing in haematology? Br J Haematol 2012;158:679-90.
3. Hofland HJ. Point of care testing and selftest related consultations in general practices in the netherlands: An exploratory study on general practitioners' experiences. Vol. Enschede: University of Twente, 2010:30.
4. Jones CH, Howick J, Roberts NW, Price CP, Heneghan C, Pluddemann A, Thompson M. Primary care clinicians' attitudes towards point-of-care blood testing: A systematic review of qualitative studies. BMC Fam Pract 2013;14:117.
5. Howick J, Cals JW, Jones C, Price CP, Pluddemann A, Heneghan C, et al. Current and future use of point-of-care tests in primary care: An international survey in australia, belgium, the netherlands, the uk and the USA. BMJ Open 2014;4:e005611.
6. National Institute of Biomedical I, Bioengineering/National Heart L, Blood Institute/National Science Foundation Workshop F, Price CP, Kricka LJ. Improving healthcare accessibility through point-of-care technologies. Clin Chem 2007;53:1665-75.
7. Cals J, van Weert H. Point-of-care tests in general practice: Hope or hype? Eur J Gen Pract 2013;19:251-6.
8. Jackman J, Uy M, Hsieh YH, Rompalo A, Hogan T, Huppert J, et al. Minding the gap: An approach to determine critical drivers in the development of point of care diagnostics. Point Care 2012;11:130-9.
9. de Vries C, Doggen C, Hilbers E, Verheij R, M IJ, Geertsma R, Kusters R. Results of a survey among gp practices on how they manage patient safety aspects related to point-of-care testing in every day practice. BMC Fam Pract 2015;16:9.
10. Heneghan C, Van den Bruel A, Thompson M, Price C, Wilson P, Crabb N, et al. Diagnostics forum 2013 report: Fast-tracking the evidence for implementing diagnostic tests. Vol. Oxford: University of Oxford, 2013:16.
11. Hopstaken RM, Kleinveld HA, van Balen JAM, Krabbe JG, van den Broek S, Weel J, et al. Richtlijn: Point of care testing (poct) in de huisartsenzorg. Vol., 2015.
