# Supplementary material for: Understanding the adoption and use of point-of-care tests in Dutch general practices using multi-criteria decision analysis
Source: BMC Fam Pract. 2019 Jan 10;20:8. doi: 10.1186/s12875-018-0893-4 (PMC6327588; doi:10.1186/s12875-018-0893-4)
Supplement: Supplementary file 2 — Questions used for semi-structured interviews, and design of analytical hierarchy process session. This file contains an overview of the interview questions used for the semi-structured interviews, as well as an overview of the design of the analytical hierarchy process session. (PDF 291 kb) [file 12875_2018_893_MOESM2_ESM.pdf]

Additional file 2: questions used for semi-structured interviews, and design of analytical hierarchy process session

This supplementary file contains an overview of the questions asked in the semi-structured interviews, as well as of the design of the analytical hierarchy process session. The interviews were conducted in Dutch. The interview questions (as shown below) were translated to English for the purpose of this publication.

Semi-structured interviews *(approximate duration: 1 hour)*

In a semi-structured interview (face-to-face or via telephone), the questions as shown below were asked to the participants. In agreement with the participants, the interviews were audio-recorded. The participants were informed that the results will be owned by the University of Twente, will only be used for scientific purposes, and will only be published or reported anonymously.

Questions with regard to the participant:

1. *What is your gender?*
2. *What is your age?*
3. *What position do you currently hold?*
4. *For how long have you already been working in this position?*
5. *In what type of organization are you employed, and where is it located?*
6. *For general practitioners only: what is the distance to the nearest central laboratory?*
7. *For general practitioners only: which point-of-care tests do you have access to, and do you use these tests frequently?*
8. *For other professionals: with which point-of-care tests are you familiar?*

Agree on the definition of point-of-care testing:

In the current Dutch guidelines, point-of-care testing within general practices is defined as [1]: "point of care testing (POCT) is the process of initiating, conducting, processing, interpreting, communicating, capturing, and follow-up of a laboratory test, by a healthcare employee, during the process of delivering healthcare to, and in near proximity of the patient.

Urine test strips, haemoglobin, and glucose measurement are classical examples of tests that can be performed point-of-care. D-dimer and C-reactive protein testing are examples of new point-of-care tests who are (gradually) finding their way to primary care.

Our current study only focuses on point-of-care tests performed within healthcare settings (i.e. within the general practice), and therefore does not include self-testing or point-of-care testing at home.

9. Question for clarification: *Do you think that a blood pressure measurement is also a point-of-care test?*
   Explanation: no, in the current study we only focus on diagnostic tests that use a sample obtained from the human body (for example, blood urine, saliva).

Questions with regard to the list of criteria, their definitions, and the range of outcome measures:

10. *Did you already study the list of criteria and their accompanying definitions? (if not, give the participants time to study this list)*
11. *Are the names of the criteria and their accompanying definitions clear, specific, and unambiguous?*
12. *Do you miss any criteria? If yes, please specify which, and why.*
13. *Do you feel that there is overlap between criteria? If yes, which criteria are this, and why? Do you feel that we could merge these criteria into one criterion?*
14. *Do you feel that there are irrelevant/redundant criteria in this list? If yes, please specify which criteria, and why.*
15. *During previous interviews, the following changes to this list were proposed* (the interviewer specifies the most important changes that were proposed during previous interviews). *How do you feel about these changes?*
16. *Do you feel that the difference between the criteria 'Technical performance', 'Positive Predictive Value', and 'Negative Predictive Value' is clear? Please explain.*
17. *For the Analytical Hierarchy Process session, it is important to determine a range for every criterion from the list. This may concern absolute numbers (e.g.: Time to perform the test: 4 minutes), or qualitative using a categorical scale (for example, for the criterion 'Connectivity': 'test result is automatically registered in the hospital information system', 'registration of the result requires an additional step (by the general practitioner)', or 'the test result is written down, and manually entered into the hospital information system'. From literature, an initial list of ranges was obtained (*which is shown to the participant*). Do you have any suggestions for improvement?*
18. *Do you have any other remarks or questions following this interview?*

References:

1. Hopstaken, R.M., et al., *Richtlijn: Point of care testing (POCT) in de huisartsenzorg*. 2015.

Analytical Hierarchy Process (AHP) session *(approximate duration: 3 hours)*

Part I. The tables below show the design of the first part of the AHP session, with the 46 pairwise comparisons in each of the four subcriteria (user, organisation, clinical value, and policy making), as well as the comparisons of these four main criteria amongst each other.

**Compare the relative importance of criterion A with criterion B, regarding the implementation of point-of-care (POC) tests in general practices**																		
**USER**																		
extremely - very strong - strong - moderate - equal - moderate -strong - very strong - extremely																		
Satisfaction patient	9	8	7	6	5	4	3	2	1	2	3	4	5	6	7	8	9	Clarity of procedure
Clarity of procedure	9	8	7	6	5	4	3	2	1	2	3	4	5	6	7	8	9	User-friendliness
User-Friendliness	9	8	7	6	5	4	3	2	1	2	3	4	5	6	7	8	9	Test interpretation
Test interpretation	9	8	7	6	5	4	3	2	1	2	3	4	5	6	7	8	9	Turn-around-time
Satisfaction patient	9	8	7	6	5	4	3	2	1	2	3	4	5	6	7	8	9	User-friendliness
Clarity of procedure	9	8	7	6	5	4	3	2	1	2	3	4	5	6	7	8	9	Test interpretation
User-friendliness	9	8	7	6	5	4	3	2	1	2	3	4	5	6	7	8	9	Turn-around-time
Satisfaction patient	9	8	7	6	5	4	3	2	1	2	3	4	5	6	7	8	9	Test interpretation
Clarity of procedure	9	8	7	6	5	4	3	2	1	2	3	4	5	6	7	8	9	Turn-around-time
Satisfaction patient	9	8	7	6	5	4	3	2	1	2	3	4	5	6	7	8	9	Turn-around-time

**ORGANISATION**																		
extremely - very strong - strong - moderate - equal - moderate -strong - very strong - extremely																		
Frequency of use	9	8	7	6	5	4	3	2	1	2	3	4	5	6	7	8	9	Room for innovation
Room for innovation	9	8	7	6	5	4	3	2	1	2	3	4	5	6	7	8	9	Workload
Workload	9	8	7	6	5	4	3	2	1	2	3	4	5	6	7	8	9	Support, training and quality control
Support, training and quality control	9	8	7	6	5	4	3	2	1	2	3	4	5	6	7	8	9	Connectivity
Frequency of use	9	8	7	6	5	4	3	2	1	2	3	4	5	6	7	8	9	Workload
Room for innovation	9	8	7	6	5	4	3	2	1	2	3	4	5	6	7	8	9	Support, training and quality control
Workload	9	8	7	6	5	4	3	2	1	2	3	4	5	6	7	8	9	Connectivity
Frequency of use	9	8	7	6	5	4	3	2	1	2	3	4	5	6	7	8	9	Support, training and quality control
Room for innovation	9	8	7	6	5	4	3	2	1	2	3	4	5	6	7	8	9	Connectivity
Frequency of use	9	8	7	6	5	4	3	2	1	2	3	4	5	6	7	8	9	Connectivity

CLINICAL VALUE																		
extremely - very strong - strong - moderate - equal - moderate -strong - very strong - extremely																		
Clinical utility	9	8	7	6	5	4	3	2	1	2	3	4	5	6	7	8	9	Technical performance
Technical performance	9	8	7	6	5	4	3	2	1	2	3	4	5	6	7	8	9	Negative predictive value
Negative predictive value	9	8	7	6	5	4	3	2	1	2	3	4	5	6	7	8	9	Positive predictive value
Positive predictive value	9	8	7	6	5	4	3	2	1	2	3	4	5	6	7	8	9	Risks
Clinical utility	9	8	7	6	5	4	3	2	1	2	3	4	5	6	7	8	9	Negative predictive value
Technical performance	9	8	7	6	5	4	3	2	1	2	3	4	5	6	7	8	9	Positive predictive value
Negative predictive value	9	8	7	6	5	4	3	2	1	2	3	4	5	6	7	8	9	Risks
Clinical utility	9	8	7	6	5	4	3	2	1	2	3	4	5	6	7	8	9	Positive predictive value
Technical performance	9	8	7	6	5	4	3	2	1	2	3	4	5	6	7	8	9	Risks
Clinical utility	9	8	7	6	5	4	3	2	1	2	3	4	5	6	7	8	9	Risks

POLICY MAKING																		
extremely - very strong - strong - moderate - equal - moderate -strong - very strong - extremely																		
Clinical guidelines	9	8	7	6	5	4	3	2	1	2	3	4	5	6	7	8	9	Scientific evidence
Scientific evidence	9	8	7	6	5	4	3	2	1	2	3	4	5	6	7	8	9	Reimbursement
Reimbursement	9	8	7	6	5	4	3	2	1	2	3	4	5	6	7	8	9	Overall costs
Overall costs	9	8	7	6	5	4	3	2	1	2	3	4	5	6	7	8	9	Legislations
Clinical guidelines	9	8	7	6	5	4	3	2	1	2	3	4	5	6	7	8	9	Reimbursement
Scientific evidence	9	8	7	6	5	4	3	2	1	2	3	4	5	6	7	8	9	Overall costs
Reimbursement	9	8	7	6	5	4	3	2	1	2	3	4	5	6	7	8	9	Legislations
Clinical guidelines	9	8	7	6	5	4	3	2	1	2	3	4	5	6	7	8	9	Overall costs
Scientific evidence	9	8	7	6	5	4	3	2	1	2	3	4	5	6	7	8	9	Legislations
Clinical guidelines	9	8	7	6	5	4	3	2	1	2	3	4	5	6	7	8	9	Legislations

CATEGORIES																		
extremely - very strong - strong - moderate - equal - moderate -strong - very strong - extremely																		
User	9	8	7	6	5	4	3	2	1	2	3	4	5	6	7	8	9	Organisation
Organisation	9	8	7	6	5	4	3	2	1	2	3	4	5	6	7	8	9	Clinical value
Clinical value	9	8	7	6	5	4	3	2	1	2	3	4	5	6	7	8	9	Policy making
User	9	8	7	6	5	4	3	2	1	2	3	4	5	6	7	8	9	Clinical value
Organisation	9	8	7	6	5	4	3	2	1	2	3	4	5	6	7	8	9	Policy making
User	9	8	7	6	5	4	3	2	1	2	3	4	5	6	7	8	9	Policy making

Part II. The tables below show the design of the second part of the AHP session, in which the criteria are scored when the current situation (perform the test in a central laboratory) is compared to the situation in which a point-of-care (POC) analyser is available within the general practice.

**Score criterion 'x' when the current situation (central laboratory test) is compared to the new situation (POC test within general practice). Three examples:<br>1) When considering the criterion 'Satisfaction Patient', which situation do you then prefer, and to what extent?<br>2) Does the current 'Room for innovation' within the general practice stimulate the use of a central laboratory test or a POC test?<br>3) Does the criterion 'Clarity of procedure' stimulate the use of a central laboratory test or a POC test? (compare the (entire) procedure performed in case of a POC test, with the situation in which a test is requested at a central laboratory, and the processing of the results once they are received).**																			
**C-reactive protein (CRP) test**																		
extremely - very strong - strong - moderate - equal - moderate -strong - very strong - extremely																		
**USER**																		
Satisfaction patient	CRP reference test at lab	9	8	7	6	5	4	3	2	1	2	3	4	5	6	7	8	9	CRP POC test at general practice
Clarity of procedure	CRP reference test at lab	9	8	7	6	5	4	3	2	1	2	3	4	5	6	7	8	9	CRP POC test at general practice
User-friendliness	CRP reference test at lab	9	8	7	6	5	4	3	2	1	2	3	4	5	6	7	8	9	CRP POC test at general practice
Test interpretation	CRP reference test at lab	9	8	7	6	5	4	3	2	1	2	3	4	5	6	7	8	9	CRP POC test at general practice
Turn-around-time	CRP reference test at lab	9	8	7	6	5	4	3	2	1	2	3	4	5	6	7	8	9	CRP POC test at general practice
**ORGANISATION**																			
Frequency of use	CRP reference test at lab	9	8	7	6	5	4	3	2	1	2	3	4	5	6	7	8	9	CRP POC test at general practice
Room for innovation	CRP reference test at lab	9	8	7	6	5	4	3	2	1	2	3	4	5	6	7	8	9	CRP POC test at general practice
Workload	CRP reference test at lab	9	8	7	6	5	4	3	2	1	2	3	4	5	6	7	8	9	CRP POC test at general practice
Support, training, qc	CRP reference test at lab	9	8	7	6	5	4	3	2	1	2	3	4	5	6	7	8	9	CRP POC test at general practice
Connectivity	CRP reference test at lab	9	8	7	6	5	4	3	2	1	2	3	4	5	6	7	8	9	CRP POC test at general practice
**CLINICAL VALUE**																			
Clinical utility	CRP reference test at lab	9	8	7	6	5	4	3	2	1	2	3	4	5	6	7	8	9	CRP POC test at general practice
Technical performance	CRP reference test at lab	9	8	7	6	5	4	3	2	1	2	3	4	5	6	7	8	9	CRP POC test at general practice
Negative predictive value	CRP reference test at lab	9	8	7	6	5	4	3	2	1	2	3	4	5	6	7	8	9	CRP POC test at general practice
Positive predictive value	CRP reference test at lab	9	8	7	6	5	4	3	2	1	2	3	4	5	6	7	8	9	CRP POC test at general practice
Risks	CRP reference test at lab	9	8	7	6	5	4	3	2	1	2	3	4	5	6	7	8	9	CRP POC test at general practice
**POLICY MAKING**																			
Clinical guidelines	CRP reference test at lab	9	8	7	6	5	4	3	2	1	2	3	4	5	6	7	8	9	CRP POC test at general practice
Scientific evidence	CRP reference test at lab	9	8	7	6	5	4	3	2	1	2	3	4	5	6	7	8	9	CRP POC test at general practice
Reimbursement	CRP reference test at lab	9	8	7	6	5	4	3	2	1	2	3	4	5	6	7	8	9	CRP POC test at general practice
Overall costs	CRP reference test at lab	9	8	7	6	5	4	3	2	1	2	3	4	5	6	7	8	9	CRP POC test at general practice
Legislations	CRP reference test at lab	9	8	7	6	5	4	3	2	1	2	3	4	5	6	7	8	9	CRP POC test at general practice

Glycated haemoglobin (HbA1c)																			
extremely - very strong - strong - moderate - equal - moderate -strong - very strong - extremely																			
**USER**																			
Satisfaction patient	HbA1c reference test lab	9	8	7	6	5	4	3	2	1	2	3	4	5	6	7	8	9	HbA1c POC test at general practice
Clarity of procedure	HbA1c reference test lab	9	8	7	6	5	4	3	2	1	2	3	4	5	6	7	8	9	HbA1c POC test at general practice
User-friendliness	HbA1c reference test lab	9	8	7	6	5	4	3	2	1	2	3	4	5	6	7	8	9	HbA1c POC test at general practice
Test interpretation	HbA1c reference test lab	9	8	7	6	5	4	3	2	1	2	3	4	5	6	7	8	9	HbA1c POC test at general practice
Turn-around-time	HbA1c reference test lab	9	8	7	6	5	4	3	2	1	2	3	4	5	6	7	8	9	HbA1c POC test at general practice
**ORGANISATION**																			
Frequency of use	HbA1c reference test lab	9	8	7	6	5	4	3	2	1	2	3	4	5	6	7	8	9	HbA1c POC test at general practice
Room for innovation	HbA1c reference test lab	9	8	7	6	5	4	3	2	1	2	3	4	5	6	7	8	9	HbA1c POC test at general practice
Workload	HbA1c reference test lab	9	8	7	6	5	4	3	2	1	2	3	4	5	6	7	8	9	HbA1c POC test at general practice
Support, training, qc	HbA1c reference test lab	9	8	7	6	5	4	3	2	1	2	3	4	5	6	7	8	9	HbA1c POC test at general practice
Connectivity	HbA1c reference test lab	9	8	7	6	5	4	3	2	1	2	3	4	5	6	7	8	9	HbA1c POC test at general practice
**CLINICAL VALUE**																			
Clinical utility	HbA1c reference test lab	9	8	7	6	5	4	3	2	1	2	3	4	5	6	7	8	9	HbA1c POC test at general practice
Technical performance	HbA1c reference test lab	9	8	7	6	5	4	3	2	1	2	3	4	5	6	7	8	9	HbA1c POC test at general practice
Negative predictive value	HbA1c reference test lab	9	8	7	6	5	4	3	2	1	2	3	4	5	6	7	8	9	HbA1c POC test at general practice
Positive predictive value	HbA1c reference test lab	9	8	7	6	5	4	3	2	1	2	3	4	5	6	7	8	9	HbA1c POC test at general practice
Risks	HbA1c reference test lab	9	8	7	6	5	4	3	2	1	2	3	4	5	6	7	8	9	HbA1c POC test at general practice
**POLICY MAKING**																			
Clinical guidelines	HbA1c reference test lab	9	8	7	6	5	4	3	2	1	2	3	4	5	6	7	8	9	HbA1c POC test at general practice
Scientific evidence	HbA1c reference test lab	9	8	7	6	5	4	3	2	1	2	3	4	5	6	7	8	9	HbA1c POC test at general practice
Reimbursement	HbA1c reference test lab	9	8	7	6	5	4	3	2	1	2	3	4	5	6	7	8	9	HbA1c POC test at general practice
Overall costs	HbA1c reference test lab	9	8	7	6	5	4	3	2	1	2	3	4	5	6	7	8	9	HbA1c POC test at general practice
Legislation	HbA1c reference test lab	9	8	7	6	5	4	3	2	1	2	3	4	5	6	7	8	9	HbA1c POC test at general practice
