# Supplementary material for: Understanding the adoption and use of point-of-care tests in Dutch general practices using multi-criteria decision analysis
Source: BMC Fam Pract. 2019 Jan 10;20:8. doi: 10.1186/s12875-018-0893-4 (PMC6327588; doi:10.1186/s12875-018-0893-4)
Supplement: Supplementary file 3 — Results of interviews and analytical hierarchy process. This supplementary file contains an overview of the detailed results of the analysis of the analytical hierarchy process (AHP), including the weighting of the subcriteria, the group inconsistency and missing judgements during the analytical hierarchy process session, as well as the preferences regarding the subcriteria for the two alternatives (i.e. point-of-care (POC) C-reactive protein (CRP) vs. CRP in a central laboratory, as well as POC glycated haemoglobin (HbA1c) vs. HbA1c in a central laboratory). (DOCX 73 kb) [file 12875_2018_893_MOESM3_ESM.docx]

Additional file 3: results of interviews and analytical hierarchy process

This supplementary file contains an overview of the detailed results of the analysis of the analytical hierarchy process (AHP), including the weighting of the subcriteria, the group inconsistency and missing judgements during the analytical hierarchy process session, as well as the preferences regarding the subcriteria for the two alternatives (i.e. point-of-care (POC) C-reactive protein (CRP) vs. CRP in a central laboratory, as well as POC glycated haemoglobin (HbA_1c_) vs. HbA_1c_ in a central laboratory). In addition, the results of two subgroup analyses of the outcomes of the AHP session are shown.

**Table 1. Performance matrix, showing the criteria included in the AHP, their description, their range, and their relative weight.**

|  | Determinant | Description | Range |
| --- | --- | --- | --- |
| **Determinants in relation to the user** | | | |
| 1 | Satisfaction patient | Extent to which the use of the POC test is expected to improve service for the patient. | High - low satisfaction patient |
| 2 | Clarity of procedure | Extent to which the procedures for using the POC test are clearly described in protocols and/or manuals. | Clear - unclear |
| 3 | User-friendliness | Extent to which the POC test is easy to perform by the layman. | Easy to perform - difficult to perform |
| 4 | Test interpretation | Extent to which the POC test result is easy to read and the various test results are easy to interpret. | Easy to interpret - difficult to interpret. |
| 5 | Turn-around-time (TAT) | Extent to which the POC test results are instantly available. | 1 - 20 minutes (preferably <4 min.) |
| **Determinants in relation to the organisation** | | | |
| 6 | Frequency of use | Extent to which the test is used sufficiently with respect to the indication and the size of the general practice. | Often- less |
| 7 | Room for innovation | The extent to which the pressure of daily practice leaves room for innovation and a mind-set for change. | None – present |
| 8 | Workload | Extent to which the POC test can be implemented without dramatic changes in the current way of working and user's workload. | Increase, equal, decrease |
| 9 | Support, training and quality control | Extent to which the introduction, maintenance, and quality control of the POC test, as well as the training of personnel is sufficient and supported by a coordinator from the laboratory, manufacturer and general practice. | No support - coordinators from lab, manufacturer and general practice in lead for contracts and regular up-dates to provide collaboration. |
| 10 | Connectivity | Extent to which the POC test results and errors are registered in an information system (HIS) | Hand written - fully automatically registration of results/errors in HIS. |
|  |  |  |  |
| **Determinants in relation to the clinical value** | | | |
| 11 | Clinical utility | Extent to which a correct (treatment) decision, as based on the point-of-care (POC) test result, has added value in clinical outcomes. | Increase, equal, decrease of clinical outcome for the patient. |
| 12 | Technical performance | The extent to which the POC test is exact, precise, reliable and robust in the hands of the user. | Increase, same, decrease. |
| 13 | Negative Predictive Value | Negative predictive value: Proportion of negative results that are true negative, which enhances the user's ability to reliably rule out a condition. | Increase, equal, decrease. |
| 14 | Positive Predictive Value | Positive predictive value: Proportion of positive results that are true positive, which enhances the user's ability to reliably diagnose a condition. | Increase, equal, decrease. |
| 15 | Risks | The impact of a (wrong) treatment/advice based on a (wrong) test result. | Increase, equal, decrease of clinical outcome for the patient. |
| **Determinants in relation to the socio-political context** | | | |
| 16 | Clinical guidelines | Extent to which the POC test is implemented in national guidelines. | None – present |
| 17 | Scientific evidence | Extent to which the added value of the POC test is demonstrated (as compared with current practice) in scientific literature, in the right patient population and for a specific clinical pathway. | Not available - sufficient and high quality |
| 18 | Reimbursement | Is the POC test reimbursed for the general practitioner? | Not / by manufacturer / double consult (time consult > 20 min.) / MI-S3 (Innovation incentive by insurer) / standard NZA rate |
| 19 | Overall costs | Extent to which the POC test is expected to decrease costs of the health care system. | Increase, equal, decrease. |
| 20 | Legislations | Extent to which the innovation fits into existing legislations. | None – present |

**Table 2. The inconsistency during the AHP session:**

**Group inconsistency Missing judgments**

User 0.07 0/10

Organisation 0.04 2/10

Clinical value 0.01 4/10

Socio-political context 0.09 2/10

Categories 0.05 0/6

Overall: 0.05 1.6/9.2

***Figures 1a-d. The outcome of the AHP session when comparing the preference of the POC CRP test with the CRP test performed at a central laboratory, for each of the subcriteria. The grey bars represent the relative weight of the subcriteria. The squares represent the preference with regard to the POC CRP test, whereas the triangles represent the preference with regard to the CRP test performed at a central laboratory. The overall weight of the main criterion is shown in the title.***

***Figure 1a.*** *Result of the AHP analysis on the alternatives POC CRP test and the CRP test performed at a central laboratory for the main criterion ‘user’.*

***Figure 1b.*** *Result of the AHP analysis on the alternatives POC CRP test and the CRP test performed at a central laboratory for the main criterion ‘organisation’.*

***Figure 1c.*** *Result of the AHP analysis on the alternatives POC CRP test and the CRP test performed at a central laboratory for the main criterion ‘clinical value’.*

***Figure 1d.*** *Result of the AHP analysis on the alternatives POC CRP test and the CRP test performed at a central laboratory for the main criterion ‘socio-political context’.*

***Figures 2a-d. The outcome of the AHP session when comparing the preference of the POC HbA_1c_ test with the HbA_1c_ test performed at a central laboratory, for each of the subcriteria. The grey bars represent the relative weight of the subcriteria. The squares represent the preference with regard to the POC HbA_1c_ test, whereas the triangles represent the preference with regard to the HbA_1c_ test performed at a central laboratory. The overall weight of the main criterion is shown in the title.***

***Figure 2a.*** *Result of the AHP analysis on the alternatives POC HbA_1c_ test and the HbA_1c_ test performed at a central laboratory for the main criterion ‘user’.*

***Figure 2b*** *Result of the AHP analysis on the alternatives POC HbA_1c_ test and the HbA_1c_ test performed at a central laboratory for the main criterion ‘organisation’.*

***Figure 2c.*** *Result of the AHP analysis on the alternatives POC HbA_1c_ test and the HbA_1c_ test performed at a central laboratory for the main criterion ‘clinical value’.*

***Figure 2d.*** *Result of the AHP analysis on the alternatives POC HbA_1c_ test and the HbA_1c_ test performed at a central laboratory for the main criterion ‘socio-political context’.*

**Table 3a. Results of subgroup analysis of the AHP session – subgroup analysis of three GPs**

|  | |  | CRP | | HbA_1c_ | |
| --- | --- | --- | --- | --- | --- | --- |
| Determinant | | | **POC CRP** | **CRP central lab** | **POC HbA_1c_** | **HbA_1c_ central lab** |
| Determinants in relation to the user | | | **86.9%** | **13.1%** | **68.5%** | **31.5%** |
| 1 | Satisfaction patient | | 90.0% | 10.0% | 84.7% | 15.3% |
| 2 | Clarity of procedure | | 84.5% | 15.5% | 50.0% | 50.0% |
| 3 | User-friendliness | | 84.5% | 15.5% | 76.0% | 24.0% |
| 4 | Test interpretation | | 81.2% | 18.8% | 50.0% | 50.0% |
| 5 | Turn-around-time | | 90.0% | 10.0% | 90.0% | 10.0% |
| Determinants in relation to the organisation | | | **74.1%** | **25.9%** | **53.2%** | **46.8%** |
| 6 | Frequency of use | | 87.4% | 12.6% | 50.0% | 50.0% |
| 7 | Room for innovation | | 90.0% | 10.0% | 83.9% | 16.1% |
| 8 | Workload | | 83.4% | 16.6% | 38.6% | 61.4% |
| 9 | Support, training and quality control | | 24.3% | 75.7% | 23.2% | 76.8% |
| 10 | Connectivity | | 44.2% | 55.8% | 38.6% | 61.4% |
| Determinants in relation to the clinical value | | | **65.1%** | **34.9%** | **47.1%** | **52.9%** |
| 11 | Clinical utility | | 88.8% | 11.2% | 50.0% | 50.0% |
| 12 | Technical performance | | 38.6% | 61.4% | 38.6% | 61.4% |
| 13 | Negative Predictive Value | | 50.0% | 50.0% | 50.0% | 50.0% |
| 14 | Positive Predictive Value | | 50.0% | 50.0% | 50.0% | 50.0% |
| 15 | Risks | | 44.2% | 55.8% | 38.6% | 61.4% |
| Determinants in relation to the socio-political context | | | **67.5%** | **32.5%** | **49.1%** | **50.9%** |
| 16 | Clinical guidelines | | 84.0% | 16.0% | 50.0% | 50.0% |
| 17 | Scientific evidence | | 87.3% | 12.7% | 46.6% | 53.4% |
| 18 | Reimbursement | | 50.0% | 50.0% | 50.0% | 50.0% |
| 19 | Overall costs | | 90.0% | 10.0% | 51.9% | 48.1% |
| 20 | Legislations | | 50.0% | 50.0% | 44.2% | 55.8% |
| Overall preference for POC or central laboratory test | | | **69.9%** | **30.1%** | **51.2%** | **48.8%** |

**Table 3b. Results of subgroup analysis of the AHP session – subgroup analysis after excluding two experts who did not participate in the AHP group session**

|  | |  | CRP | | HbA_1c_ | |
| --- | --- | --- | --- | --- | --- | --- |
| Determinant | | | **POC CRP** | **CRP central lab** | **POC HbA_1c_** | **HbA_1c_ central lab** |
| Determinants in relation to the user | | | **83.4%** | **16.6%** | **65.6%** | **34.4%** |
| 1 | Satisfaction patient | | 89.9% | 10.1% | 85.0% | 15.0% |
| 2 | Clarity of procedure | | 77.1% | 22.9% | 46.1% | 53.9% |
| 3 | User-friendliness | | 83.4% | 16.6% | 75.1% | 24.9% |
| 4 | Test interpretation | | 75.7% | 24.3% | 50.0% | 50.0% |
| 5 | Turn-around-time | | 90.0% | 10.0% | 90.0% | 10.0% |
| Determinants in relation to the organisation | | | **64.2%** | **35.8%** | **49.2%** | **50.8%** |
| 6 | Frequency of use | | 88.5% | 11.5% | 50.0% | 50.0% |
| 7 | Room for innovation | | 89.9% | 10.1% | 84.6% | 15.4% |
| 8 | Workload | | 81.3% | 18.7% | 29.8% | 70.2% |
| 9 | Support, training and quality control | | 23.7% | 76.3% | 26.4% | 73.6% |
| 10 | Connectivity | | 37.1% | 62.9% | 45.7% | 54.3% |
| Determinants in relation to the clinical value | | | **52.8%** | **47.2%** | **44.1%** | **55.9%** |
| 11 | Clinical utility | | 89.3% | 10.7% | 50.0% | 50.0% |
| 12 | Technical performance | | 31.0% | 69.0% | 36.1% | 63.9% |
| 13 | Negative Predictive Value | | 47.8% | 52.2% | 50.0% | 50.0% |
| 14 | Positive Predictive Value | | 47.8% | 52.2% | 50.0% | 50.0% |
| 15 | Risks | | 36.9% | 63.1% | 34.7% | 65.3% |
| Determinants in relation to the socio-political context | | | **71.1%** | **28.9%** | **51.9%** | **48.1%** |
| 16 | Clinical guidelines | | 85.4% | 14.6% | 50.0% | 50.0% |
| 17 | Scientific evidence | | 87.1% | 12.9% | 59.8% | 40.2% |
| 18 | Reimbursement | | 58.2% | 41.8% | 50.0% | 50.0% |
| 19 | Overall costs | | 89.9% | 10.1% | 53.4% | 46.6% |
| 20 | Legislations | | 45.4% | 54.6% | 42.6% | 57.4% |
| Overall preference for POC or central laboratory test | | | **61.6%** | **38.4%** | **48.8%** | **51.2%** |
